# Radar-based remote physiological sensing: Progress, challenges, and opportunities

**DOI:** 10.3389/fphys.2022.955208

**Published:** 2022-10-11

**Authors:** Shekh Md Mahmudul Islam

**Affiliations:** Department of Electrical and Electronic Engineering, University of Dhaka, Dhaka, Bangladesh

**Keywords:** Min.5-Max. 8, non-contact sensing, biomedical radar sensor, physiological sensing, breathing rate (BR), heart rate

## Abstract

Modern microwave Doppler radar-based physiological sensing is playing an important role in healthcare applications and during the last decade, there has been a significant advancement in this non-contact respiration sensing technology. The advantages of contactless, unobtrusive respiration monitoring have drawn interest in various medical applications such as sleep apnea, sudden infant death syndromes (SIDS), remote respiratory monitoring of burn victims, and COVID patients. This paper provides a perspective on recent advances in biomedical and healthcare applications of Doppler radar that can detect the tiny movement of the chest surfaces to extract heartbeat and respiration and its associated different vital signs parameters (tidal volume, heart rate variability (HRV), and so on) of the human subject. Additionally, it also highlights the challenges, and opportunities of this remote physiological sensing technology and several future research directions will be laid out to deploy this sensor technology in our day-to-day life.

## Introduction

There is a growing need for continuous monitoring of the vital signs of humans for assessing their health status (Massaroni et al., 2019). Two different types of modalities such as contact sensors (finger pulse, chest belt) and non-contact sensors (Radar and Camera) are used for monitoring different vital signs such as breathing rate and heart rate (Massaroni et al., 2020). Contactless remote sensing of vital signs is gaining more attention than contact sensors as it is unobtrusive and users need not intentionally engage themselves with the system (Molinaro et al., 2022). Non-Contact and unobtrusive monitoring of human cardiopulmonary activity is one of the promising solutions for remote respiratory monitoring of COVID patients, sleep apnea diagnosis, post-surgery monitoring, in-home elderly people fall detection, and life signs detection under debris for post-disaster search and rescue applications (Broic-Lubecke et al., 2012; Li et al., 2013; Islam et al., 2021; Li et al., 2021). One of the potential advantages of this technology is that without attaching any sensor to the body it could facilitate the health monitoring of lonely elderly patients in home settings, enable sleep monitoring of patients without any expensive sleep laboratory, and detect the trapped injured person under rubble (Broic-Lubecke et al., 2012; Li et al., 2013; Li et al., 2021). Among the non-contact sensing modalities, Radar seems more promising than a camera as it is privacy-invasive and no imaging technology is required for vital signs sensing (Li et al., 2021). Table 1 illustrates the comparison between the Radar and Camera for vital signs monitoring. For vital signs extraction using the camera is mostly performed from the intensity of the light and the phase information of the reflected echoes of the Radar can also extract vital signs. Radar is mostly employed for military and defense applications such as detecting aircraft and ships but last few decades it has been modified for biomedical applications (Islam, 2020). Commercially available radar system such as 2.4 GHz–24 GHz Frequency modulated continuous wave (FMCW) RADAR is available in the market and can be used for vital signs monitoring (Islam et al., 2022a). Doppler radar remote respiration sensing technology has shown potential in several applications and demonstrated proof of concept in numerous disease assessments such as obstructive sleep apnea (OSA), Sudden Infant Death Syndrome (SIDS), and COVID-19 as well (Li et al., 2013; Islam et al., 2021). The efficacy of this remote respiration sensing technology has also been proven during the COVID-19 pandemic time as it can track the status of breathing remotely without attaching any sensor to the body (Islam et al., 2021).

**TABLE 1:** A comparison of Camera and Radar System for Vital Signs Monitoring.

Contactless sensing system	Methods for vital signs monitoring	Advantages	Disadvantages
Camera (Canon)	The intensity of reflected light	•Higher accuracy	•Privacy issue
Radio-Frequency (RF) (Radar/WiFi-Router)	Reflected signal phase information	•Unobtrusive•No line of sight is required	•Sensitive to any other random motions
		•Post-processing is faster
		•Privacy-invasive

The traditional method for respiratory monitoring is the electrocardiogram (ECG) which is carried out with the attachment of electrodes to the chest surface. The attachment of the sensor to the body provides skin irritation and is not suitable for long-term continuous monitoring (Broic-Lubecke et al., 2012; Li et al., 2013). Long-term monitoring of cardiorespiratory activity of patients with sleep disorders, ill elderly patients, premature infants, and burn-victim patients is essential without the attachment of any sensor to the body. Long-term unobtrusive respiratory monitoring can help to prevent cardiac arrest and it can reduce mortality as well. Therefore, bringing this unobtrusive respiratory monitoring technology into the real world can help to reduce the mortality rate and different vital signs can be monitored continuously without wearing any sensor on the body (Broic-Lubecke et al., 2012).

This unobtrusive and non-contact respiration sensing technology can play a significant role beyond healthcare applications such as occupancy detection in an in-home environment and counting the number of people for energy savings buildings (Yavari et al., 2014), finding trapped or injured people during post-disaster search and rescue applications (Islam ShekhM. M. et al., 2020), vital sign monitoring remotely of COVID-19 patients (Massaroni et al., 2020), sleep posture recognition (Islam et al., 2022b) and authenticating people from the radar-captured cardiopulmonary signatures (Islam ShekhMdMahmudul et al., 2020). This perspective article concentrates on discussing the recent advances in radar-based non-contact sensing technology, especially discussing different vital signs parameter extraction processes. It will also highlight the latest advancement of this technology for deployment in different application areas and its associated challenges. The core contribution of this work is as follows:

• This short article demonstrates a perspective of using Radar for vital signs monitoring and its associated different parameters that can also be used for health status assessment.
• The article also highlights different research challenges associated with this non-contact sensing technology and its advancement in different new research avenues.

Overall, the goal of this work is to provide a brief perspective of this technology to the readers so that especially young researchers can also engage themselves to work in this area considering the broader spectrum of this technology.

## Basic principle of non-contact sensing technology and extracted vital signs

**FIGURE 1:**
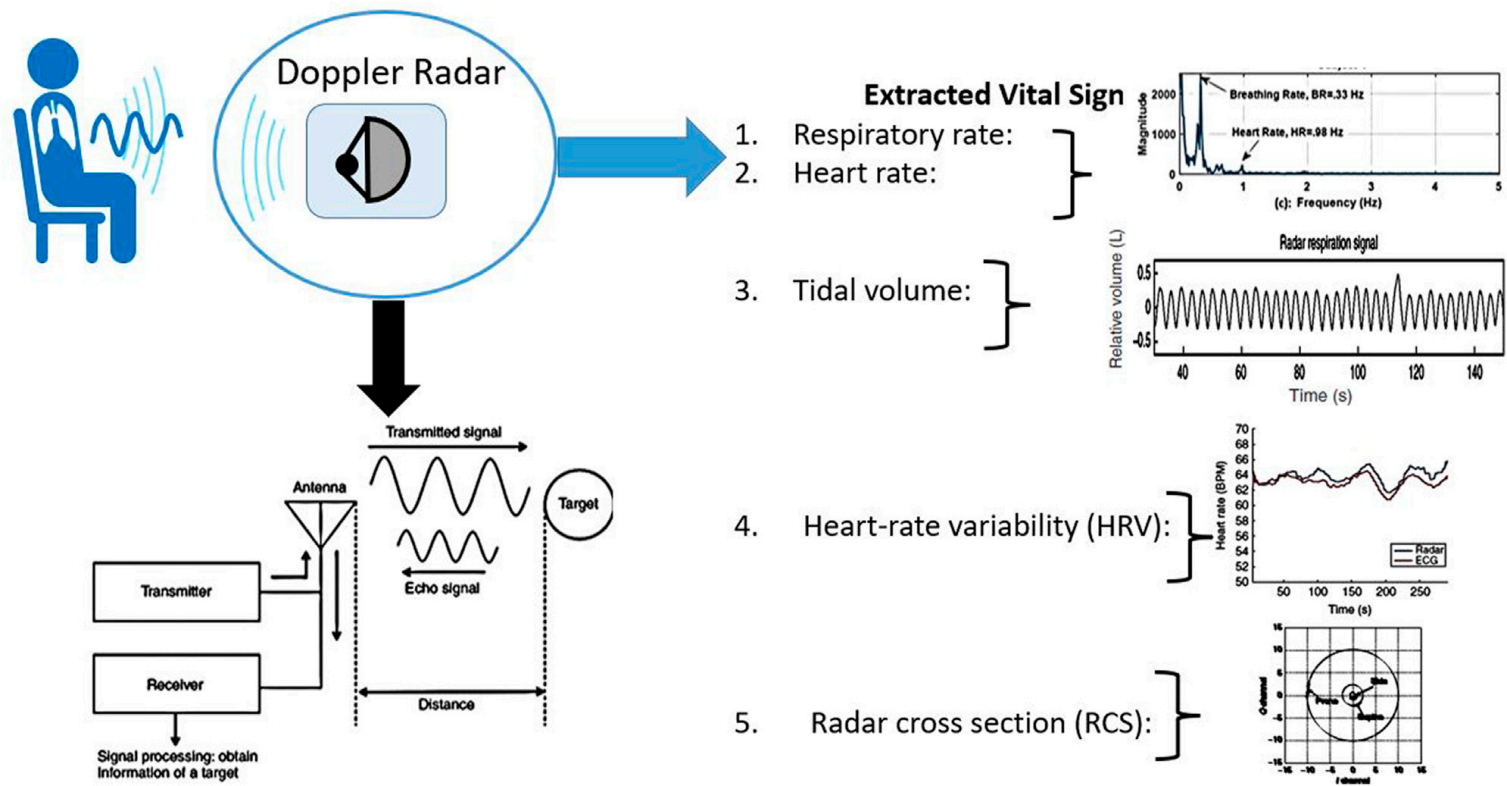
The basic principle of Radar-based non-contact physiological sensing. A typical Radar sends an electromagnetic signal and from the reflected echo tiny movements of the human chest’s surface can be tracked. Different vital sign parameters can also be extracted including breathing rate, heart rate, tidal volume, heart rate variability, and Radar cross-section (RCS). Radar circuitry components’ typical block diagram is also shown in the figure.

The fundamental principle of non-contact sensing technology is that the Radar sends an electromagnetic signal toward humans and when the signal is reflected its phase changes. The phase change of the reflected signal is directly proportional to the tiny movement of the chest surface due to cardiorespiratory activities (Gu et al., 2012). “RADAR” stands for radio detection and ranging and it consists of a transmitter and a receiver shown in Figure 1. Radar may have the same antenna both for transmission of the electromagnetic signal and reception of the reflected echoes. After capturing the reflected echoes different signal processing is employed for extracting different vital signs such as breathing rate, heart rate, tidal volume, heart rate variability (HRV), and Radar cross-section (RCS) shown in Figure 1. Different vital sign parameters can also be extracted from the Radar-reflected echoes and they are described below.

### Respiratory rate

Respiratory rate (RR) is one of the critical vital signs that need to be tracked regularly for monitoring personal health information (Broic-Lubecke et al., 2012). However, due to the limitation of the currently used measuring devices the measurement of respiratory rate often goes underestimated. A stable and constant respiratory rate is an indicator of good quality of health. On the other hand, a sudden change in RR can also predict an imminent health crisis (Broic-Lubecke et al., 2012). Respiratory rate can be extracted from the radar reflected echoes by performing filtering of the signal within the range 0.001–0.4 Hz and then taking the fast Fourier transform (FFT) of the filtered signal (Broic-Lubecke et al., 2012). In the literature, it also has been demonstrated that there is a good correlation between the Doppler radar and respiratory chest belt for extracting respiratory rates obtained from healthy persons (Droitcour, 2006; Broic-Lubecke et al., 2008). The human study was also carried out using radar at different medical centers; the respiratory rate was extracted in subjects with lung conditions and obstructive sleep apnea; the accuracy of the reported system was greater than 98.25% (Broic-Lubecke et al., 2012). Indoor activity and vital signs monitoring during different activities is also possible using the FMCW radar module as Radar can provide the range, velocity, and angle information of humans (Yang et al., 2021).

### Heart rate

One of the important vital signs such as heart rate can also be extracted from the radar reflected echo after striking the chest surfaces of a human. The signal processing steps are quite similar to extracting respiratory rate from radar signals. After capturing the radar reflected echoes it needs to be bandpass filtered within 0.8-0.2 Hz and then FFT analysis needs to be performed for extracting heart rate (Broic-Lubecke et al., 2012). In literature, different studies were reported and the radar measured the heart rate of healthy persons was also compared with the reference Electrocardiogram (ECG) and finger pulse sensor (Broic-Lubecke et al., 2008; Broic-Lubecke et al., 2012). All the reported studies demonstrated that there is a good correlation between the radar-measured heart rate and the reference sensor measurement (Broic-Lubecke et al., 2008; Broic-Lubecke et al., 2012). Traditional methods for measuring heart rate are all contact sensor-based and are gaining popularity but are still challenged in terms of having their clinical significance established.

### Tidal volume

Tidal volume is the amount of air that is inhaled or exhaled during one breathing cycle (Massagram et al., 2009). It has been proven that there is a linear relationship between the volume of the air inhaled or exhaled during the breathing cycle and the chest wall displacement (Massagram et al., 2009). Currently to measure lung volume or airflow typically face masks or mouthpieces are used (Massagram et al., 2009). The use of face masks or mouthpieces is always obtrusive and it can also change the respiration pattern of the subject. Another indirect measurement technique is the use of thermocouples or capnography. A thermocouple is used intermittently to measure the changes in nasal temperature during breathing and can also extract information on tidal volume. On the other hand, capnography measures the concentration of the partial pressure of carbon-di-oxide ( $CO_{2}$ ) using an oral-nasal cannula for measuring tidal volume. Both thermocouple and capnography require the placement of the sensor on the body which is cumbersome and uncomfortable. As there is a linear relationship between the chest wall displacement and the air volume changes, the measurement of the movement of the chest should be proportional to the tidal volume. Therefore, Doppler radar can also extract the tidal volume of the human without attaching any sensor to the body. Tidal volume can be calculated from the peak to peak amplitude of the volume displacement (Massagram et al., 2009; Massagram et al., 2013). Different studies were also performed for measuring the accuracy of the Radar for extracting tidal volume. It has been reported that the correlation coefficient between the spirometer and radar measurement is 0.95378 which indicates that tidal volume can also be measured continuously without attaching any sensor to the body (Massagram et al., 2009).

### Heart rate variability

Heart rate variability (HRV) refers to the beat-to-beat changes in heart rate that indicates the activity of automatic regulation of how the cardiovascular system works. HRV analysis has received significant attention for evaluating the effectiveness of the autonomic nervous system (Boric-Lubecke et al., 2008). Continuous measurement of the beat-to-beat interval of the respiration waveform is used to measure the heartbeat which is expressed as beats per minute. The traditional method of continuous measurement of HRV is the electrocardiogram (ECG) where beat-to-beat intervals are always monitored. ECG measurement is always cumbersome as the human subject needs to wear this sensor to the chest surfaces. Many new bio-logging ECG devices can measure R-R intervals using finger-based sensors such as the pulse wave method where photoplethysmography (PPG) or piezoresistive sensors are mostly used where patients need to be tethered to the sensing devices (Alqaraawi et al., 2016). Remote respiration monitoring using microwave Doppler radar can also be used for the detection of HRV as it can acquire the beat-to-beat intervals within the window such as from 2 to 5 min and for short-term study this window has been selected and then verified with the long-term clinical study for a window of more than an hour (Boric-Lubecke et al., 2008; Massagram et al., 2009). Thus, the remote monitoring of HRV could be a powerful tool for different disease assessments such as obstructive sleep apnea (OSA), sudden infant death syndrome (SIDS) and also providing burn victim care. The reported results also demonstrated that the Doppler radar can also extract beat-to-beat intervals with acceptable accuracy in comparison with the other established methods.

### Radar Cross Section (RCS) for measuring the shape/orientation of the subject

The RCS is a measure of the wave reflecting from the target and therefore, the shape or orientation of the target can be estimated from the reflected echoes of the radar signal. RCS is also measured by calculating the power density scattered from the target with the incident power density (Kiriazi et al., 2021, Islam et al., 2022). The RCS of a target can be measured by comparing the received power with the transmitted power and some studies were performed for estimating the received power loss by integrating propagation loss formulas such as Friis propagation loss (Kiriazi et al., 2021). For cardio-respiratory monitoring of human RCS of the moving tiny movement of the chest can also be extracted and known as cardiopulmonary effective radar cross section (ERCS). For a quadrature biomedical Doppler radar receiver, there are two channels for acquisition of the signal one is the in-phase channel signal (I channel) and another one is the quadrature-phase channel signal (Q channel). The baseband signal I-Q plot provides an arc if the motion of the target is periodic and from the arc, the center can be estimated. The radius of the arc is directly proportional to the ERCS (Kiriazi et al., 2021, Islam et al., 2022). From the reflected echoes ERCS sleep postures such as supine, prone, and side can be recognized (Kiriazi et al., 2021, Islam et al., 2022). Therefore, biomedical Doppler radar has been demonstrated for automatic sleep posture recognition and the quality of sleep can also be determined (Kiriazi et al., 2021, Islam et al., 2022). This particular non-contact sensing technology can be a potential hand-held device for sleep disorder diagnosis and intervention. Table 2 below illustrates the comparative accuracy analysis of the Radar sensor with the traditional sensor for vital signs parameter extraction.

**TABLE 2:** A comparative analysis of different vital signs parameters extracted by Radar and its accuracy with the traditional sensor.

Parameters	Reference sensor	Radar sensor details	Field of applications	Results
Breathing rate (BR)	Chest belt	2.4 GHz/24 GHz Radar Continuous wave (CW)	Continuous Breathing monitoring	Accuracy: 98.25% (Boric-lubecke et al., 2012)
Heart rate (HR)	Finger pulse sensor	2.4 GHz CW Radar	Continuous Heart rate monitoring	Accuracy: 97.52% (Boric-Lubecke et al., 2008)
Tidal volume	Spirometer	2.4 GHz CW Radar	Airflow profile measurement	Accuracy: 95.4% (Massagram et al., 2009)
Heart rate variability (HRV)	ECG/Fingertip sensor	2.4-GHz CW/FMCW Radar	Cardiac disease assessment	Accuracy: 95.24% (Boric-Lubecke et al., 2008)
Radar Cross Section (RCS)	Chest belt	2.4/24-GHz CW/FMCW Radar	Sleep quality (Posture, apnea, and so on)	Accuracy: 96.23% (Kiriazi et al., 2021)

## Scopes for new research avenues

Radar-based non-contact physiological sensing technology has been applied to healthcare applications for more than 4 decades. Recently, this non-contact sensing technology is being used in different application areas especially being employed with different communication technologies such as WiFi or cellular networks. Integration of WiFi and other conventional communication technologies also created new research avenues for non-contact sensing technology. New research avenues are discussed below.

### Hand gesture and sleep posture recognition

Radar has been used for different human gestures and posture recognition. This particular application has opened a new era of human-computer interaction (HCI) as this approach is non-contact and unobtrusive. The use of miniaturized radar for gesture recognition has been investigated for several decades and recently Google has integrated a miniaturized radar module into the Google Pixel phone for controlling phone buttons without attaching the surface of the phone (Ahmed et al., 2021). Additionally, people also investigated utilizing radar for different sleep posture recognition (Islam et al., 2022). The goal of this project is to propose a non-contact sleep posture recognition system for sleep interventions and different sleep disorder assessments. Further exploration can bring this technology into the real world of applications.

### Emotion recognition

Another interesting new research avenue is emotion recognition from the radio-frequency (RF) signals reflected from the body. From the reflected echo different key features are extracted to recognize numerous emotions such as sadness, happiness, and so on (Zhao et al., 2016; khan et al., 2021; Pradhan et al., 2017; Rattanyu and Mizukawa, 2011). Radar technology has been used to extract different heart-beat-related parameters so just extracting heart rate-related features helps to recognize different emotions. One of the potential aspects of this research area is this non-contact sensor technology can directly measure the interaction of emotions and physiological signals without attaching any sensor to the body.

### Animal monitoring

Remote vital signs and activity monitoring of animals in the natural environment can provide valuable information such as behavioral patterns, thermoregulation, energy expenditure and also health status. In general, the metabolic state of the animal is measured by injecting radio-labeled water and then observing the rate of carbon-di-oxide ( $C0_{2}$ ) production in several weeks (Speakman and Racey, 1986). The method involves enriching the body water of a subject with heavy hydrogen (^2^H) and heavy oxygen (^18^O) and then determining the difference in washout kinetics between both isotopes, being a function of carbon dioxide production (Speakman et al., 1986) As this existing technique depends on the biological half-life of oxygen so measuring the activity of animals is always cumbersome. As this existing technique depends on the biological half-life of oxygen so measuring the activity of animals is always cumbersome. The main drawback of the doubly layered water technique is mass spectroscopy is required to measure the washout kinetics between both isotopes which is expensive and required a good amount of time for assessment (Speakman et al., 1986). Doppler radar motion-sensing technology can provide a better tool for the automated activity monitoring of animals as it can sense different motions accurately and therefore, an active investigation is going on for animal monitoring (Singh et al., 2012). One potential benefit of this sensing technology is that no mass spectroscopy is required and isotope intake is not also not required as the method is non-contact and unobtrusive (Singh et al., 2012).

## Future challenges

A growing interest in utilizing radio frequency (RF) in healthcare applications has paved the way for the development and commercialization of the miniaturized radar and there has been a significant advancement both in hardware and signal processing areas. However, still there remain significant challenges that need to be addressed to bring this sensor technology into the real world of applications. The associated challenges for the scopes of new research avenues are highlighted below.

### Radar physiological sensing in multi-subject environments

Radar-based non-contact physiological sensing in multi-subject environments always remains a significant challenge as when there is a presence of multiple subjects in front of the radar system it gets a combined mixture of respiration patterns (Islam et al., 2021). Therefore, isolating independent respiratory signatures from the combined mixture always remains a technological challenge for the implementation of this sensor technology in the real world (Islam et al., 2022). Recent research demonstrated that integrating blind source separation techniques such as independent component analysis (ICA) with the radar system can help to solve this particular problem. Additionally, the direction of arrival (DOA) estimation technique can also help to isolate the respiratory pattern from the combined mixture of respiration patterns (Islam et al., 2022). Recognizing people from their breathing diversity using a non-contact sensor is gaining attention and different respiratory kinetics-related features have been extracted from radar-captured respiratory patterns such as inhale/exhale area ratio, area depth, and so on. An initial study has been performed on more than twenty subjects and showed a reasonable accuracy for recognizing people from their radar captured breathing diversity (Islam S. M. M. et al., 2020; Rahman et al., 2018; Islam et al., 2021, Islam et al., 2022). Active investigation and exploration are ongoing for isolating the respiratory patterns from the combined mixture because in reality there is a high chance of the presence of multiple subjects in front of the radar system for different applications such as identity authentication, occupancy sensing, and so on.

### Random body movement cancellation

Vital signs monitoring in real life always remains a significant challenge because there is a high chance of the presence of random body movements during tracking the tiny movements of the chest surfaces. Mitigation of random body movement during vital signs monitoring remains a significant challenge for bringing this technology into the real world of applications (Cardillo et al., 2021). One of the potential approaches is the use of the matched filter to retrieve the respiration and heartbeat-related spectra from the combination of respiration signals with random body movement. People also attempted to integrate the phase compensation technique at the Doppler RF front end and also in the baseband signal for mitigating the random body movement during vital signs monitoring (Cardillo et al., 2021). With the continued investigation this existing research problem needs to be resolved to bring this sensor technology into the real world of applications.

### Radar-based identity authentication data security

Traditional authentication systems such as passwords and facial recognition using intrusive video imaging have several drawbacks especially none of them are continuous and privacy-invasive. Therefore, researchers are investigating whether it is possible to recognize people from their breathing patterns that will be captured unobtrusively. From the radar-reflected echoes, people attempted to find a unique respiratory-related biomarker for recognizing people without attaching any sensor to the body (Rahman et al., 2018; Islam et al., 2021; Islam et al., 2022). The initial feasibility study of the proposed non-contact identity authentication has been performed and further exploration is going on to bring this technology into the real world of applications. As one of the new research avenues is to utilize the biomedical Doppler radar for identity authentication. Data security of the proposed radar-based identity authentication system also remains a challenge as radar data may be falsified or different false information may be injected into the radar data. Therefore, designing and integrating different security protocols with the radar-based identity authentication system can help to mitigate the mismanagement of the radar-based identity authentication system (Islam et al., 2020).

## Conclusion

Remote respiration sensing using microwave Doppler radar has shown great promise not only in health care applications but also in different human-machine interaction areas as well. Last 4 decades there has been a significant advancement in this technology for remote monitoring of vital signs and other security applications also. This non-contact life-sensing technology has brought significant benefits to human society. This article highlights some of the recent research avenues of this technology and associated research challenges for bringing this sensor technology into the real world of applications. The author also wishes this perspective article could not only serve as a resource for both researchers and engineers but also provide a direction to the recent research trends and areas that need to be explored for pushing forward this technology for different promising applications.

## Data Availability

The original contributions presented in the study are included in the article/supplementary material, further inquiries can be directed to the corresponding author.
